# Generalized fault diagnostics of polymer electrolyte fuel cells using machine learning

**DOI:** 10.1016/j.isci.2025.113350

**Published:** 2025-08-13

**Authors:** Greg D’Silva, Eashaal Mahmood, Rhodri Jervis, Shangwei Zhou

**Affiliations:** 1 Electrochemical Innovation Lab, Department of Chemical Engineering, University College London, WC1E 7JE London, UK

**Keywords:** Electrochemical energy production, Machine learning, Engineering

## Abstract

Polymer electrolyte fuel cells (PEFCs) are promising for mobile and stationary applications, but short operational lifetimes and frequent faults limit their commercial viability. This work introduces a black-box diagnostic method using multifrequency Walsh function perturbation signals to detect water management and starvation faults. The approach improves signal-to-noise ratio and accuracy without harming the cell. Using voltage response as the diagnostic variable, dense neural networks (DNNs), 1D convolutional neural networks (1D-CNNs), and support vector machines (SVMs) were tested. All models accurately classified normal, drying, and starvation conditions in a single PEFC, with 1D-CNN and SVMs reaching 100% accuracy. However, model generalization to a different PEFC was poor. Including data from multiple PEFCs significantly improved performance, with the 1D-CNN showing superior generalization, even when trained on limited unseen data. This establishes the 1D-CNN as the most effective model for robust, scalable PEFC diagnostics across varied datasets.

## Highlights

• Walsh signals used for high signal-to-noise ratio fuel cell diagnosis
• Combined MEA data improves generalization to unseen fuel cells
• 1D-CNN shows strong performance using only 20% data from the unseen MEA

## Graphical abstract

**Figure:**
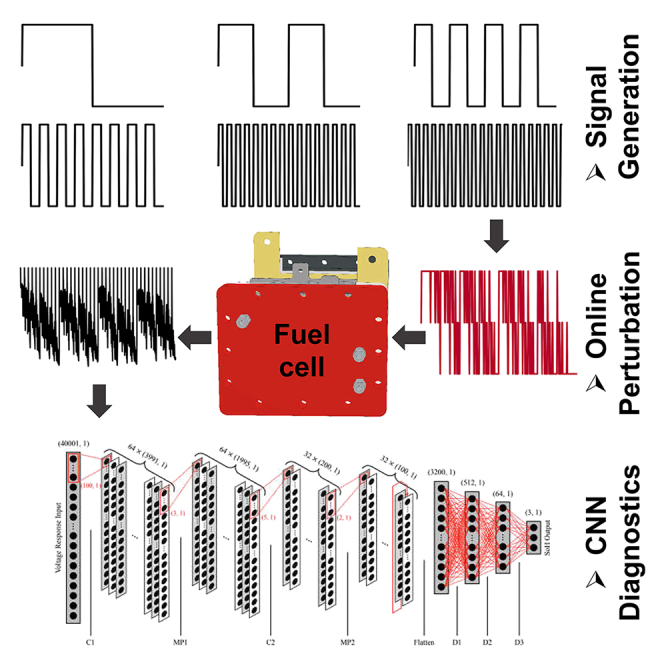

## Introduction

As the global population rises, a fundamental shift away from fossil fuels is urgently required to satisfy the increasing energy demand of the modern world. Not only is the extraction and combustion of fossil fuels detrimental to the environment due to their associated greenhouse gas emissions, but sources of oil, coal, and natural gas are becoming increasingly expensive and difficult to extract. Polymer electrolyte fuel cells (PEFCs) have the potential to play a crucial role in solving some of these issues, producing no harmful emissions during their operation. They are cleaner and more efficient than traditional combustion engines. However, one of the largest factors limiting the commercial ubiquity of PEFCs is their high likelihood of faults. For the operation to remain stable, PEFCs must be controlled within a narrow range of operating conditions; deviation from these conditions leads to enhanced degradation and performance losses.^1^ Although some faults can be corrected via control action (recoverable faults), certain faults can only be rectified via the complete replacement of components (permanent faults). Water management is a critical issue for PEFCs, accounting for over 50% of all failures and can be divided into flooding and drying faults.^2^ Drying reduces the ionic conductivity of the membrane and, in addition to performance decline, can also result in permanent membrane degradation since it becomes more brittle and prone to cracks and pinholes.^3^ Hence, it should be noted that if recoverable faults are not detected in time, their status will exacerbate, often leading to permanent faults and damage to the materials in the fuel cell. There is a clear motivation for efficient fault diagnosis strategies to identify and rectify faults before they irreversibly worsen.

Fault diagnostic strategies can be classified into offline and on-line methods. Offline methods involve experiments that take place under laboratory conditions and may require disassembly of the PEFC.^1^ While these techniques are especially useful in understanding the fundamental characteristics and behaviors of PEFCs, their intrusive nature makes them inappropriate for onboard, real-time use in operational cells. In contrast, on-line diagnosis techniques only use (often crude) signals from the sensors attached to the PEFC to estimate its state of health (SoH). Although this means that measurements can be taken more quickly and continuously compared to offline techniques, much less physical information on the SoH of the PEFC is realized with each measurement. Therefore, computational models are integrated to assist in the diagnostic procedure, improving the accuracy of SoH estimations and providing meaning to the sensor data.^4^ On-line techniques can be classed as model-based (white-box), data-driven (black-box), or a combination of the two (grey-box).

In model-based fault diagnosis, a mathematical model representing the real process runs alongside the PEFC during operation. Consisting of theoretical differential and/or algebraic equations, the process model takes the same input conditions received by the operating PEFC to generate an output. The model output is then compared with the output data of the PEFC, and the difference between the two “residual” is calculated; a residual of zero indicates normal operation, while a non-zero residual signifies the presence of a fault. In 2009, Escobet et al.^5^ modified the MATLAB/Simulink PEFC model developed by Pukrushpan et al.^6^ to include a set of six different fault states. The faults were implemented into the model via the sensitivities of the residuals, allowing the SoH to be identified based on the relative values of the model residuals. The fault regions could then be displayed in the three-dimensional residual space, with the health of the PEFC being predicted based on the Euclidian distance between the position of the system itself and the six different faults. While this demonstrates how a white-box model can successfully identify faults in real-time, it is too computationally demanding to be applied to a real system due to the large number of model equations involved. Moreover, since the model is a representation of the physical system, a perfectly accurate model simply cannot exist; hence, the model uncertainty must also be considered, further increasing its overall complexity.^7^ In the future, advances in onboard computing power may increase the viability of white-box models as a means for fault identification; however, as they stand, they are too complex to implement on-line.

Due to the intricacy of white-box models, black-box models are becoming increasingly popular due to their simplicity and speed. Rather than being based on fundamental physical equations, black-box models are derived from experimental datasets and diagnose faults via machine learning algorithms, which classify the experimental data into different health states. However, this limits their genericity compared to their white-box counterparts, whose model parameters can be easily adjusted to reflect other PEFCs; instead, black-box models require a large historical dataset from the exact/similar PEFC to function effectively. One of the most widely used black-box models is the artificial neural network (ANN), consisting of nodes or “neurons” organized into layers with the nonlinear activation function, through which information can pass and be classified.^8^

The use of neural networks in fuel cell diagnostics has increased in the last 15 years or so. In 2010, Yousfi Steiner et al.^9^^,^^10^ attempted to identify water management issues in PEFCs using a recurrent neural network (RNN), whereby the outputs of some neurons are fed back to previous layers, facilitating the storage of time-series data. When a fault occurred in the PEFC, its output deviated from that of the RNN, generating a residual and allowing the fault state to be detected. Although the RNN modeled the system well, its ability to be generalized to other PEFC systems was significantly reduced since it was fitted too heavily to the exact PEFC used in the experiments. In 2013, to improve the issue of generalization, Shao et al. used an ANN ensemble to identify four different faults associated with the cooling and reactant delivery systems.^11^ As proposed by Hansen and Salamon, ANN ensembles consist of multiple sub-ANNs, which are trained separately before combining their outputs to form an ensemble output.^12^ This, therefore, improves their generalization capability, since each sub-ANN can be trained on a different physical variable of the PEFC, preventing overfitting to any specific variable. When testing its performance, the ANN ensemble achieved a diagnostic accuracy of 93.24%, representing a significant improvement over the accuracies of the sub-ANNs alone, which ranged in accuracy from 75.24% to 85.62%. Support vector machines (SVMs) are a more recently developed subset of machine learning algorithms which formulate a hyperplane in a multidimensional space and classify data depending on which side of the hyperplane it lies.^13^ It was applied to PEFC fault diagnosis in 2016 by Li et al.,^14^ who developed an on-line platform to predict SoH in real time based on the cell voltage profiles of cells within a stack. Attaining a global classification accuracy of 84.98%, it was concluded that the main reason for reductions in accuracy was due to the time delay of the on-line model. Furthermore, since the model was pre-trained on laboratory data, it did not account for the aging effect of the PEFC; long-term performance degradation could cause the cell voltages to become too dissimilar from those used to train the model, reducing diagnostic accuracy.

Electrochemical impedance spectroscopy (EIS) is a widely used diagnostic technique in electrochemical systems and involves the perturbation of the operating state using a sinusoidal current/voltage and recording the amplitude and phase of the corresponding voltage/current response. The perturbation signal frequencies range from around 0.1 Hz to 10 kHz, such that processes acting over a wide range of timescales can be explored,^15^ though the measurements must be carried out sequentially and thus EIS has a time period of several minutes for measurement. Recently, we combined all the testing frequencies into a single multifrequency signal via summation to solve the long measurement time problem associated with EIS.^16^ This was then used to perturb the PEFC, with the corresponding AC (alternating current) voltage response being measured. Traditionally, the system response is converted to the frequency domain and analyzed with Nyquist plots; however, in this case, the data were kept in the time domain for two main reasons. Firstly, all the information available in the Nyquist plots is also available in the AC voltage response of the system, albeit heavily convoluted. Secondly, conversion to the frequency domain is often time-consuming and computationally demanding; to diagnose the system as quickly as possible, removing this stage of the process helped significantly. The AC voltage response was used directly as the diagnostic variable for the input to a 1-dimensional convolutional neural network (1D-CNN), which was trained with samples corresponding to flooding, drying, and normal operating states, producing a diagnostic accuracy of 100%. Despite this, a drawback of the methodology is that the summation of the perturbation signals increases the overall amplitude of the multifrequency signal. Typically, a perturbation amplitude of 5%–10% of the DC current is used since excessive amplitudes alter the operation of the PEFC, while diminutive amplitudes are hidden in operating noise, reducing measurement accuracy.^17^

A recent study,^18^ which followed up on the proposed AC voltage response, improved diagnostics by selecting key frequency points via the distribution of relaxation times. This work takes a step further and focuses on developing a methodology to diagnose PEFC faults in a fast, on-line and nonintrusive manner to maintain optimal operation and improve durability. The final objective is for these efforts to help realize the commercial ubiquity of PEFCs, providing a reliable and clean alternative energy source for use in mobile or stationary applications.

Elsewhere, recent work has explored methods to improve the generalizability of PEMFC fault diagnostic methods to reduce the reliance on extensive fault data. Gong et al.^19^ developed a digital twin platform to simulate fault data, which was used to train a temporal convolutional network (TCN). This learning was transferred to a different fuel cell system via a domain adaptive transfer convolution network (DATCN), achieving 98.5% accuracy. While accurate, this method relies on sufficient similarity between the digital and physical systems, which cannot always be guaranteed. Chen et al. utilized long short-term memory (LSTM) networks to identify insulation faults in fuel cell vehicles.^20^ Again, their model demonstrated a high accuracy of 99.91%; however, its application was fault specific, limiting its broader use.

In this work, we demonstrate that utilizing the Walsh function to construct multifrequency perturbation signals can effectively maximize the signal-to-noise ratio between the AC voltage response and normal operating noise. For the same perturbation signal amplitude, the Walsh function can generate a larger PEFC voltage response in comparison to the summation method, solving the issue of excessive perturbation amplitude faced in previous work.^16^ We also demonstrate the generalization capabilities of the 1D-CNN algorithm, in comparison to other neural network and SVM models, by training and testing it on two physically different membrane electrode assemblies (MEAs).

## Results

### Signal generation and acquisition

To perform the simultaneous multifrequency perturbation technique as outlined by Zhou et al.,^16^ a Reference 3000 potentiostat/galvanostat (Gamry Instruments, USA) was used to apply AC perturbations to the PEFC and record the corresponding voltage response, with a sampling frequency of 10 kHz. Multifrequency perturbation signals with durations of 4 s (any data after the first 1 s is just for extra and repetitive information) were applied, followed by a 4 s period to allow the PEFC to return to its steady state operation. This was repeated 200 times (1,600 s) for each SoH, with the AC voltage responses used directly as the inputs for diagnostic machine-learning algorithms.

**Figure 1:**
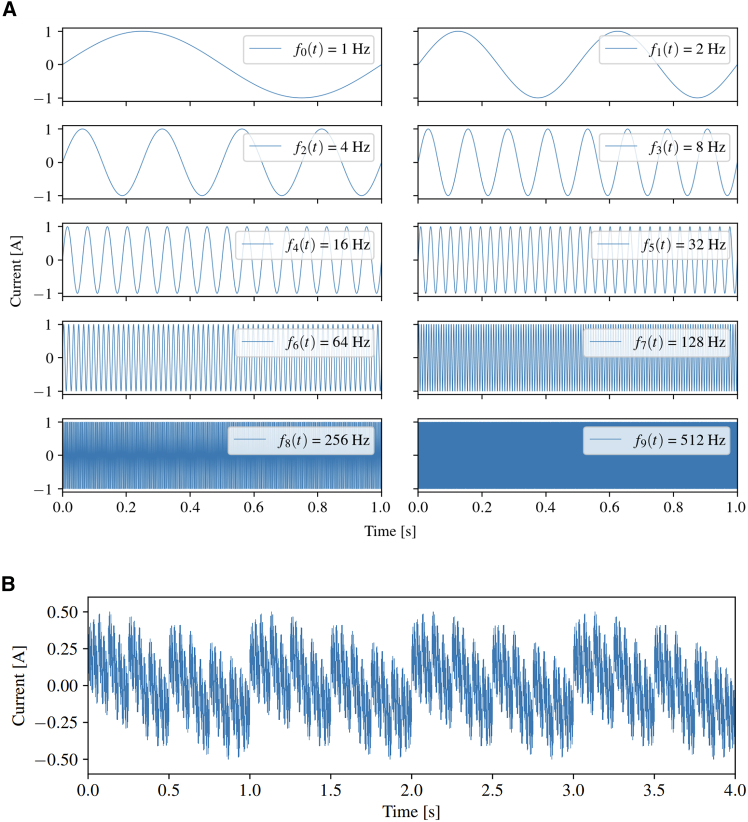
Construction of multifrequency perturbation signal using multisine summation (A) Individual AC signals ranging from 1 Hz to 512 Hz used to construct the multifrequency perturbation signal via multisine summation. (B) Final multifrequency perturbation signal. The maximum amplitude of 0.5 A corresponds to 5% of the nominal 10 A DC current.

Two different methods were used for the formulation of the AC perturbation signals. First, the summation method used by Zhou et al. was investigated,^16^ whereby the signals of each frequency are simply added together to obtain one multifrequency signal. To probe processes of different time scales within the PEFC signals across a spectrum spanning from 1 Hz to 512 Hz were used, as shown in Figure 1A. In this way, the range of testing frequencies used in EIS (such that processes acting over a wide range of timescales can be explored) is combined into one multifrequency signal. Hence, we are unable to produce a Nyquist plot; however, all the information available in the Nyquist plot is also available in the PEMFC voltage response, albeit heavily convoluted. This complexity presents an opportunity to leverage machine learning algorithms, which can extract this convoluted information that is invisible to the human eye. High-frequency signals are included to probe short-time scale processes (e.g., ohmic resistance) while low-frequency signals are included to probe long-time scale processes (e.g., mass transport resistance) such that no key frequency bands are omitted.

Denoting these frequencies as $f_{k}\left(t\right)$, the multifrequency signal $f_{M}\left(t\right)$ can be expressed as:

(Equation 1) $$\begin{array}{cc} f_{M}\left(t\right)=K\cdot \sum_{k}{\;f}_{k}\left(t\right)\text{,} & k\in \left\{0,\ldots ,9\right\}\text{,} \end{array}$$

(Equation 2) $$\begin{array}{cc} \text{where}\;f_{k}\left(t\right)=\sin\left(2^{k}2\pi t\right)\text{,} & k\in \left\{0,\ldots ,9\right\} \end{array}$$

The amplitude of $f_{M}\left(t\right)$ is controlled by the scaling constant K. The perturbation amplitude was set at 5% of the operating current; at 400 mA cm^−2^ with an active area of 25 cm^2^, the current is 10 A, so the maximum amplitude of $f_{M}\left(t\right)$ was set at 0.5 A, as shown in Figure 1B. The second method used was based on the Walsh function (WF), which generates a square binary signal according to Equation 3:

(Equation 3) $$f_{k}\left(t\right)=\text{sgn}\left(\sin\left(2^{k}2\pi t\right)\right)$$

The signum function sgn(x) is a piecewise function defined as follows:

(Equation 4) $$\text{sgn}\left(x\right)=\left\{ \begin{array}{ll} -1, & if\;x<0\\ 0, & if\;x=0\\ 1, & if\;x>0 \end{array}\right.$$

**Figure 2:**
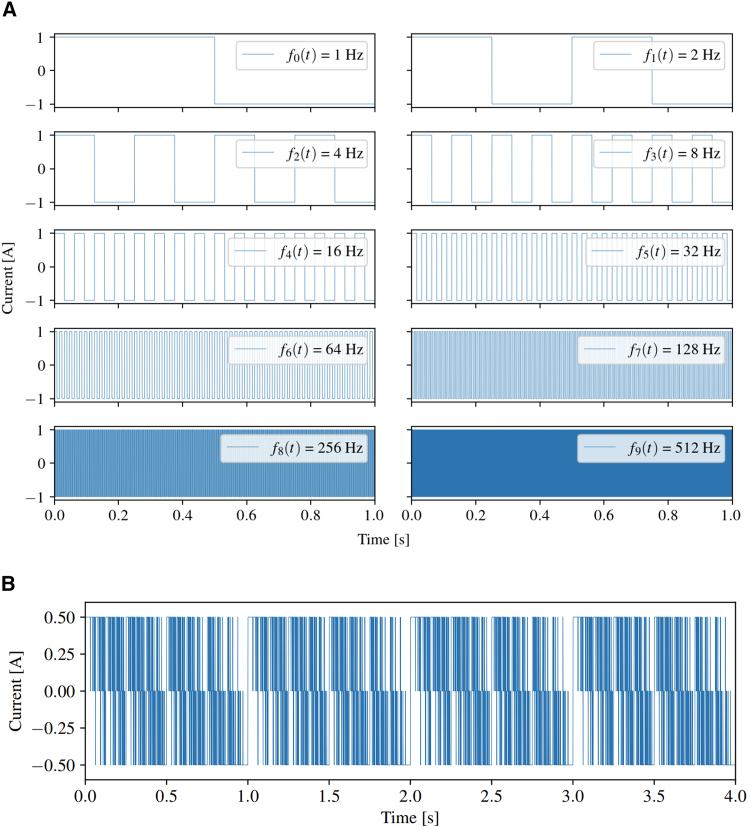
Construction of multifrequency perturbation signal using Walsh function method (A) Individual AC signals ranging from 1 Hz to 512 Hz used to construct the multifrequency perturbation signal via the Walsh function method. (B) Final multifrequency perturbation signal obtained using the Walsh function method.

Hence, the 10 individual WF frequencies from 1 Hz to 512 Hz can be visualized as in Figure 2A. The multifrequency WF signal can then be formulated via Equation 5, summing the individual frequencies and passing them through another signum function:

(Equation 5) $$\begin{array}{cc} f_{M}\left(t\right)=K\cdot \text{sgn}\left\{\sum_{k}\text{sgn}\left\{\sin\left(2^{k}2\pi t\right)\right\}\right\}\text{,} & \;k\in \left\{0,\ldots ,9\right\} \end{array}$$

The scaling constant K can then be used to adjust the amplitude to 0.5 A, yielding the signal displayed in Figure 2B.

### Diagnostic algorithms

The AC voltage responses were used directly as the diagnostic variables for SoH predictions; this was achieved by passing the voltage responses as inputs to three machine learning algorithms: dense neural networks (DNNs), one-dimensional convolutional neural networks (1D-CNNs), and support vector machines (SVMs) using linear and radial basis function (RBF) kernel functions. Further details can be found in the Data S1: Diagnosis algorithms. DNNs were used as they can model complex, non-linear relationships in high-dimensional data, which aligns well with the nature of the AC voltage responses; however, they can be prone to overfitting when data are limited.

**Figure 3:**
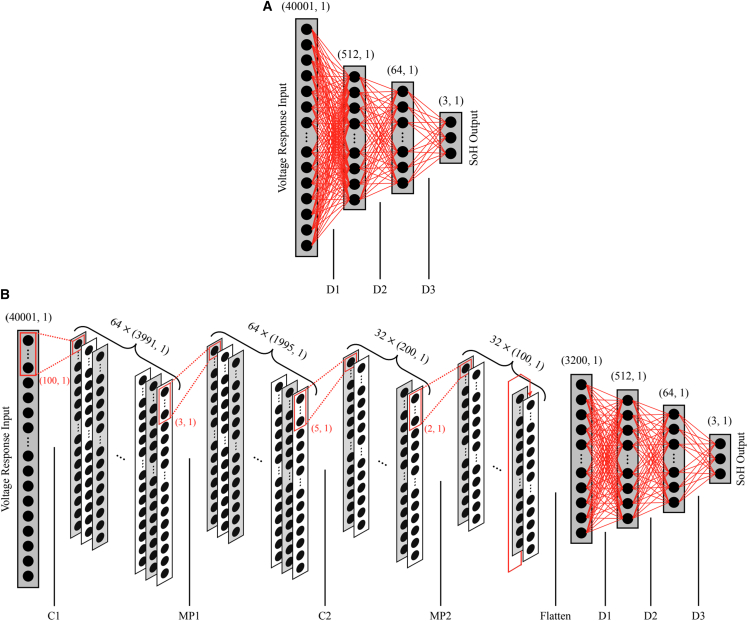
Architectures of the DNN and 1D-CNN models used for PEMFC fault classification (A) Schematic of the DNN structure showing layer types and dimensions. D, dense layer. (B) Schematic of the 1D-CNN structure. Convolutional and max pooling layers reduce input dimensionality and extract local features. C, convolutional layer; MP, maxpool layer.

The DNN was built using TensorFlow 2.15.0^21^ in Python 3.10.2 according to the structure outlined in Table 1 and Figure 3A. 1D-CNNs, on the other hand, are well-suited for capturing spatial patterns in sequential data, such as those found in the voltage responses, potentially reducing the chance of overfitting. The 1D-CNN was also built using TensorFlow according to the specifications in Table 2 and Figure 3B. Both networks were trained via the minimization of their cross-entropic losses as outlined in Equation S5. SVMs were included for their memory efficiency, as they calculate optimal decision hyperplanes without relying on the full dimensionality of the input space. This is advantageous given the high information content and large size of the AC voltage responses; however, their performance is known to degrade with highly non-linear data. The SVM models were implemented using scikit-learn 1.2.2^22^ in Python 3.10.2. Additional details regarding the architecture of these algorithms are provided in the supplemental information.

**Table 1:** Properties of each layer of the fully connected deep neural network

Layer	Dimension	Parameters	Activation
Input	(40001, 1)	–	–
D1	(512, 1)	20,481,024	ReLU
D2	(64, 1)	32,832	ReLU
D3	(3, 1)	195	Softmax

The input layer has a size of 40001 since the voltage response was used directly as the input to the network and was recorded for 4 s by the Gamry potentiostat at a sampling rate of 10 kHz (10,000 samples per second). The output layer has a size of 3 since the predicted health state is either normal, drying, or starvation. D, dense layer.

**Table 2:** Properties of each layer of the 1-dimensional convolutional neural network

Layer	Dimension	Parameters	Activation	Filters	Filter Size	Filter Stride
Input	(40001, 1)	–	–	–	–	–
C1	(3991, 64)	6,464	ReLU	64	(100, 1)	10
MP1	(1995, 64)	–	–	64	(3, 1)	2
C2	(200, 32)	10,272	ReLU	32	(5, 1)	10
MP1	(100, 32)	–	–	32	(2, 1)	2
Flatten	(3200, 1)	–	–	–	–	–
D1	(512, 1)	1,638,912	ReLU	–	–	–
D2	(64, 1)	32,832	ReLU	–	–	–
D3	(3, 1)	195	Softmax	–	–	–

C, convolutional layer; MP, max pool layer; D, dense layer. The output of the convolutional layers is flattened before it can be passed into the fully connected layer since it has a higher dimensionality from the number of filters used.

### Perturbation signal comparison

**Figure 4:**
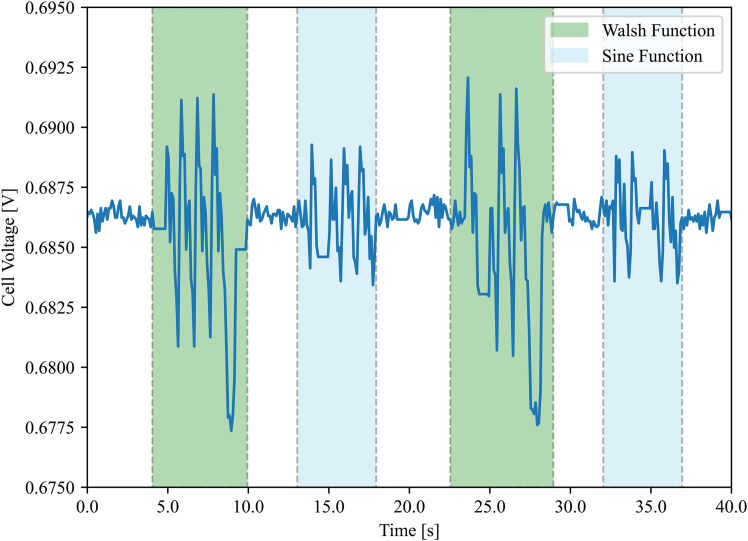
Comparison of cell voltage response to multisine and Walsh function perturbations Sample of the cell voltage response during alternating AC perturbations using multisine and Walsh function signals.

To compare the two types of perturbation signals, the PEFC was operated under normal conditions while applying AC perturbations, alternating between the multisine and WF signals from Figures 1B and 2B, respectively. A 40 s sample of the cell voltage can be seen in Figure 4, highlighting the regions where each perturbation was applied.

Figure 4 highlights that, for the same perturbation amplitude, the WF signal is able to generate a response in the cell voltage that is approximately two times larger than that generated by the multisine signal; it is, therefore, easier to differentiate between the WF response and the normal voltage noise of the PEFC. In comparison, the low signal-to-noise ratio of the multisine signal can result in measurement errors when passed through subsequent machine learning (ML) algorithms, since it becomes harder to distinguish between the AC voltage response and the normal voltage noise, as discussed by Giner-Sanz et al.^23^ Obtaining a similar response amplitude with the multisine method would require an increase in the perturbation signal amplitude. However, this is detrimental since the system needs to be maintained in a steady state; thus, the perturbation amplitude must be large enough to avoid measurement errors but small enough to prevent deviation from the normal operation. The improvement in signal-to-noise ratio was investigated in previous work by Zhou et al.,^24^ who analyzed the fast fourier transform frequency spectra of both the WF and multisine signals. It was found that the WF signal had nearly twice the amplitude for each frequency. Therefore, the WF perturbation is superior to the multisine signal since, for the same amplitude, it can generate a larger voltage response in the PEFC, solving the issue of excessive perturbation amplitude faced in previous work.^16^

### Fault diagnostic procedure

**Figure 5:**
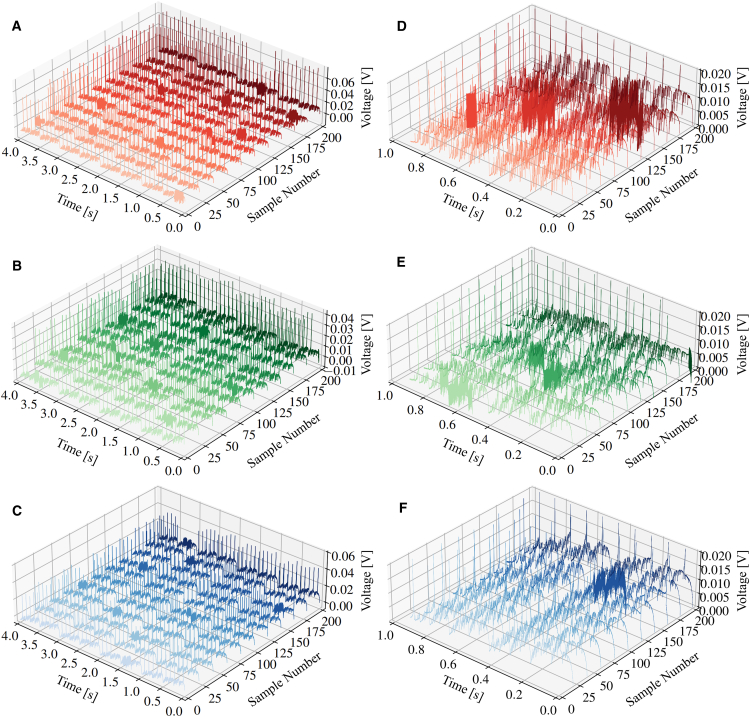
Normalized voltage responses under different operating conditions for ML diagnostics (A–C) Normalized voltage responses used as diagnostic inputs under (A) drying, (B) normal, and (C) starvation conditions. While 200 responses were collected per condition, 11 representative samples are shown for clarity. (D–F) Locally enlarged sections of the voltage responses for (D) drying, (E) normal, and (F) starvation conditions, highlighting the rich diagnostic information embedded in the signal features.

The potentiostat was used to apply the perturbation signals 200 times to the PEFC during each fault state. Since the variation of the voltage is the variable of concern (i.e., the response to the applied perturbation), each voltage sample was subtracted from the first value in the sequence. This has the effect of eliminating the DC voltage component, leaving behind the AC component, and also serves to normalize the data, transforming the voltage responses to be on a similar scale of ±0.1 V to improve the training stability and performance of any subsequent ML models.^25^ Hence, the voltage responses were produced for all three fault states as displayed in Figure 5, ready to be passed directly into ML algorithms as the diagnostic variables for classification.

Although the perturbation signal remained unchanged for each sample, the AC voltage responses were observed to be different under different health states. However, for samples within the same health state, the voltage responses remained similar. Figure 5 shows the drying, normal, and starvation voltage responses, as well as locally enlarged versions to identify the subtle differences between health states. Looking at the locally enlarged plot for the normal state, the voltage response remains at approximately +0.005 V for all samples with less variability. In contrast, for the drying and starvation states, the responses are more erratic and increase rapidly to over +0.010 V. While these subtle differences between the voltage responses can be noticed by the human eye, neural networks and other ML algorithms that excel at pattern recognition can utilize all of the rich information stored within the responses, instantly and accurately distinguishing between them.

### Performance of different algorithms

The hypothesis was that the 1D-CNN would perform best due to its unique ability to extract spatially correlated features from the information-rich voltage responses. The DNN was expected to be more prone to overfitting and computationally intensive due to the larger number of parameters involved in its architecture. However, it facilitates a direct comparison to the 1D-CNN, highlighting the effectiveness of its convolutional layers. Finally, the SVM was hypothesized to achieve high accuracy if the voltage responses in the feature space were clean and well separated but struggle if noisy and overlapping.

**Figure 6:**
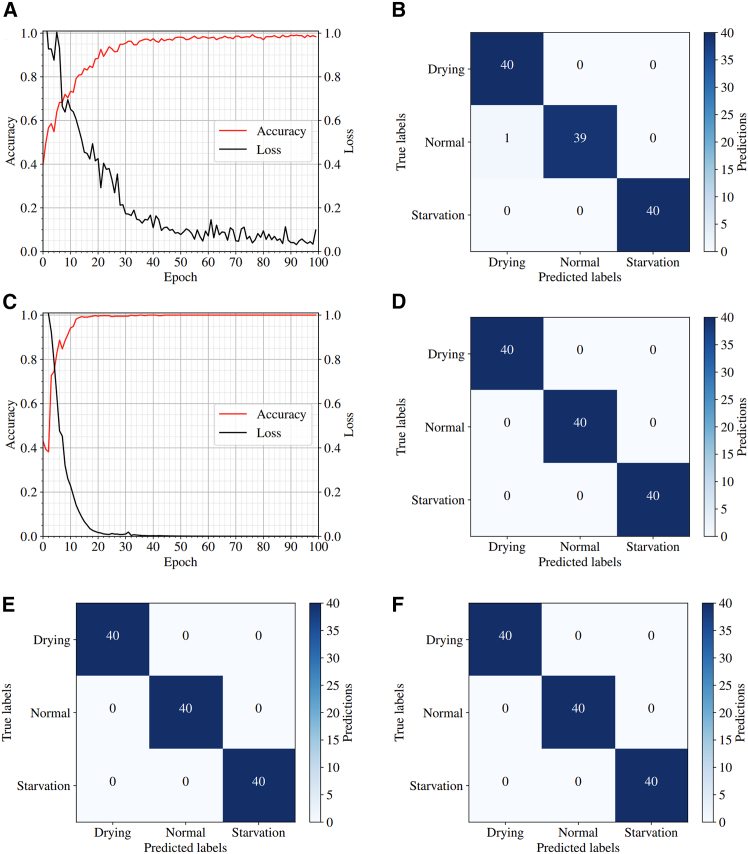
Training performance and classification accuracy of ML models for PEMFC diagnostics (A) Training accuracy and loss curves for the DNN. (B) Confusion matrix for DNN classification results, with correct predictions along the principal diagonal. (C) Training accuracy and loss curves for the 1D-CNN. (D) Confusion matrix for 1D-CNN test results. (E) Confusion matrix for the SVM classifier with a linear kernel. (F) Confusion matrix for the SVM classifier with a radial basis function kernel.

The voltage responses were divided into a train-test split of 80:20, whereby 480 random responses were used to train the DNN, and the remaining 120 were used to evaluate its accuracy. The diagnostic results can be seen in Figures 6A and 6B. Despite the large number of parameters to be trained, after 100 complete passes through the training data (epochs), the DNN reached a training accuracy and loss of 98.38% and 0.0983, respectively, demonstrating that the network was able to successfully converge and diagnose the SoH based on the AC voltage response. However, the instability and fluctuations during training are typical indicators of underfitting, where the network struggles to correctly predict the unseen data as the parameters have not been fully optimized.^26^ Nevertheless, the DNN was able to converge and performed well on the test data, achieving a diagnostic accuracy of 99.17%, only misclassifying one of the 120 test voltage responses, as revealed in Figure 6B, where the rows of the confusion matrix indicate the actual SoH while the columns indicate the predictions. Due to the stochastic nature of the Adam optimizer, random initialization of the network parameters, and random train-test partition, the diagnosis results may vary slightly between executions.^27^

The diagnostic accuracy of the 1D-CNN was also investigated, repeating the same procedure as before. Figure 6C indicates that the 1D-CNN converged faster and more smoothly than the DNN during training, suggesting that the optimized parameters fit the data more closely. Unlike the DNN, where the neurons between each layer are fully connected, the convolutional layers connect each neuron to a region of neurons in the previous layer, reducing the number of connections in the model.^28^ The 1D-CNN possesses ∼12 times fewer parameters than the DNN, as indicated by Tables 1 and 2, reducing the computational demand on the optimizer. This is an important factor for on-board and real-time diagnosis in operating systems. In addition, the convolutional and pooling layers within the network reduce the dimensionality of the AC voltage response input, extracting spatially correlated features. The dense layers are then used to diagnose the SoH based on this condensed feature map, rather than the entire voltage response as in the DNN, simplifying the optimization task and reducing convergence time.^29^ The 1D-CNN reached a training accuracy and loss of 100% and 2.291 × 10^−4^, respectively, indicating a slight improvement over the DNN, and achieved a diagnostic test accuracy of 100% as shown in Figure 6D.

The diagnostic procedure was also repeated for the SVM models with linear and RBF kernels. Figures 6E and 6F demonstrate that both kernels were effective in classifying the 120 testing samples, achieving diagnostic accuracies of 100%. In general, SVM is known to perform efficiently in high-dimensional spaces; in this case, the dimension of the voltage responses is R^40001,^ and the SVM models were still able to construct the classification hyperplanes with ease. Another key advantage of SVM is its memory efficiency since it only needs the set of support vectors to make a prediction rather than the whole dataset.^30^ The large number of parameters in the neural networks makes them relatively memory inefficient in comparison. However, as mentioned previously, perfect diagnostic accuracy suggests that there are clear margins of separation between each SoH class; SVM is known to perform poorly when the training data have more noise; hence, when additional data are passed to the SVM model from a different MEA or operating condition, its accuracy could be significantly reduced if there is any overlap between the datasets. This issue was investigated further by Sabzekar et al.,^31^ who used fuzzy SVMs to differentiate between outliers and reduce the effect of noisy data; however, this was not explored here.

### Generalization procedure

To test the generalization capability of the models, an additional identical MEA was fabricated, and the fault procedure was repeated to obtain a further 200 voltage responses for normal, drying, and starvation states. Thus far, the voltage responses were all taken from the same MEA at a similar point in its life; as the PEFC is operated over its lifespan, its performance will change accordingly, potentially altering the way it responds to the multifrequency perturbation signals and rendering the previously trained model obsolete. Hence, it is paramount to test if the model can predict the health states of PEFCs operating outside the training data range rather than being constrained to the original data.

The rationale behind repeating the data collection on a different MEA was to explore how the model deals with data from a different cell that is also performing “normally”. The polarisation curves in Figure S2 were employed to compare the performances of the MEAs before commencing the testing procedure. These indicate that the unseen MEA had a better performance, achieving 39.0% and 61.9% higher power and current densities, respectively; since the unseen MEA had a different performance, it could be used effectively to investigate the generalization capability of the models. The datasets from each MEA can be defined as follows:

$$D_{1}=\text{Old}\;\text{MEA}\;\text{voltage}\;\text{responses}$$

$$D_{2}=\text{Unseen}\;\text{MEA}\;\text{voltage}\;\text{responses}$$

**Figure 7:**
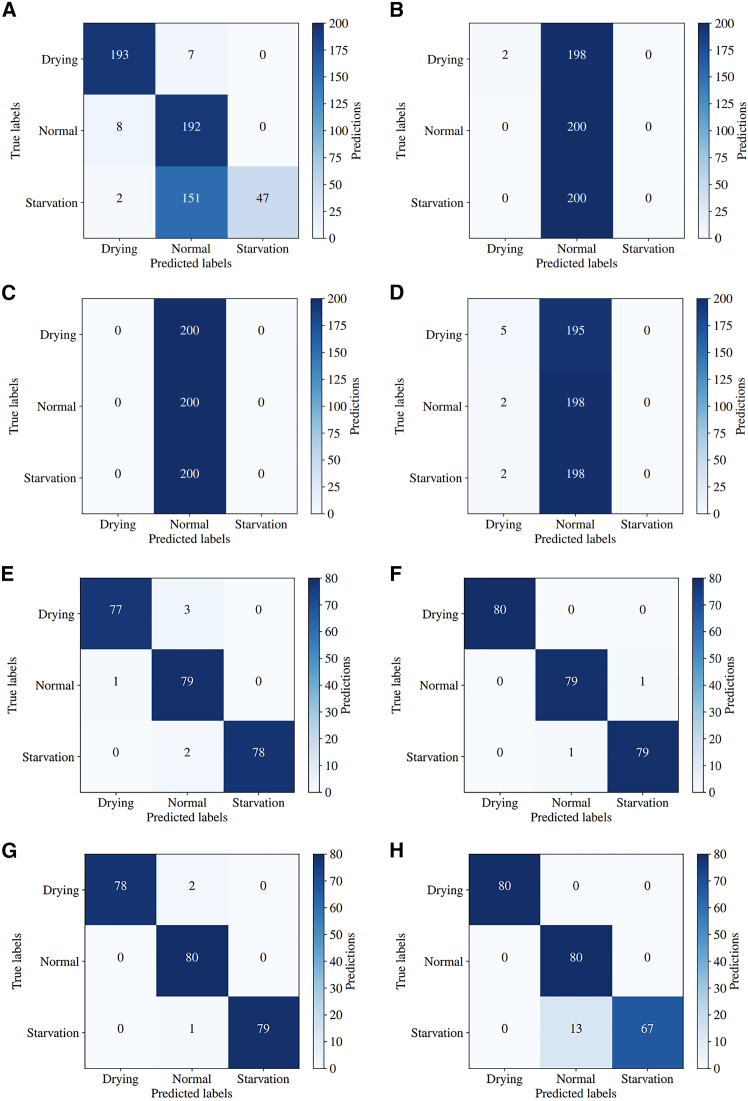
Generalization test confusion matrices for ML models using different training and test datasets (A–D) Confusion matrices showing model performance when trained on dataset D_1_ and tested on dataset D_2_ for (A) DNN, (B) 1D-CNN, (C) linear-SVM, and (D) RBF-SVM. (E–H) Confusion matrices after combining datasets D_1_ and D_2_ into a single dataset, with models tested on 80% of this combined data: (E) DNN, (F) 1D-CNN, (G) linear-SVM, and (H) RBF-SVM.

The algorithms were independently trained on D_1_ and tested on D_2_ to check if they could predict the SoH of an MEA that the model had never “seen” before. The diagnostic accuracies can be seen in Figures 7A–7D, indicating that the DNN outperformed the other models. Although the DNN was not able to fit as closely to the original data initially, this improved its generalisation capability, predicting 72% of the unseen data correctly. Conversely, when tested on D_2_, the 1D-CNN and SVMs only predicted ∼33% of the unseen data correctly. Since there are three target classes, this accuracy indicates that the models were not able to distinguish between any of the fault states between the two datasets.

To improve the generalization performance, datasets $D_{1}$ and $D_{2}$ were combined into one rich dataset to give the following train-test splits:

$$\text{Training}\;\text{data}=\alpha \left(D_{1}+D_{2}\right)\text{,}$$

$$\text{Testing}\;\text{data}=\left(1-\alpha \right)\left(D_{1}+D_{2}\right)\text{,}$$

where α is the train split fraction set at 0.8. Thus, the models were trained using 960 random samples from $D_{1}$ and $D_{2}$, and were tested using the remaining 240 samples. From Figures 7E–7H, it is evident that the diagnostic accuracy for all models was improved by incorporating data from both MEAs into the training set. This ensured that the models were fitted to the health states of both MEAs, rather than being constrained to one, as observed in Figures 7A–7D. The 1D-CNN and linear-SVM performed similarly well, achieving diagnostic accuracies of 99.2% and 98.8%, respectively. The DNN achieved a slightly lower accuracy of 97.5% while the RBF-SVM performed notably worse, misclassifying 13 starvation samples as normal.

Although these results suggest that it is necessary to train the models using data from both MEAs, this is not ideal as, for the model to predict the SoH of any generic MEA, it’s possible it would require previous data from the same MEA (though it could also be the case that training data containing a sufficiently large number and variation of MEAs would be able to classify unseen MEAs that are outside of the training dataset. This is impractical for several reasons, including the excessive amount of data required to train the models and the fact that, if an MEA changes unexpectedly during its lifetime, the model may no longer be able to predict its SoH, though more extensive investigations are required at this time to confirm this. Furthermore, the experimental results were obtained at the specified operating conditions in Table 3; if the same MEA was operated at a different current density, for example, there is no guarantee that the model will perform successfully at conditions that it was not trained with. This highlights the main limitation of black-box models, being their dependence on a good-quality historical dataset, which is often difficult or impossible to obtain.

**Table 3:** Operating conditions required for normal, air starvation, and drying health states

Condition	Current density (mA cm^−2^)	Inlet temperatures (°C)	Anode stoichiometry	Cathode stoichiometry
Normal	400	52.5	+1.5	+3.0
Starvation	400	52.5	+1.5	+1.5
Dehydration	400	40.0	+1.5	+3.0

The cell was heated to 60°C in all cases.

**Figure 8:**
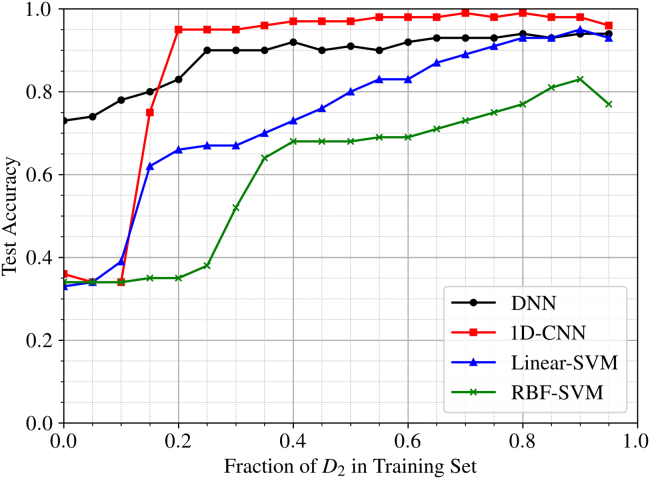
Generalization test accuracies with incremental inclusion of dataset D_2_ in training Model accuracies when trained on dataset D_1_ supplemented with varying proportions of dataset D_2_, showing the effect of added data on generalization performance.

One potential solution to the issue of requiring a large training dataset is to reduce the number of samples from each individual MEA. In this way, a few samples from a wide variety of different MEAs at different points in their life and different operating conditions could be used, such that any normally operating MEA will fit somewhere into this range. This would avoid having to train with data from every MEA that makes use of the model, provided that the initial testing data are taken from an extensive range of MEA ages and operating conditions. To test the concept of only including a small amount of unseen data from a different MEA, the train and test data were set up as follows:

$$\text{Training}\;\text{data}=D_{1}+\beta D_{2}\text{,}$$

$$\text{Testing}\;\text{data}=\left(1-\beta \right)D_{2}\text{,}$$

where β is the fraction of D_2_ included in the training set. Varying β from 0 to 0.95 reveals the amount of D_2_ that needs to be present in the training set for the model to accurately predict the SoH. In essence, the algorithms were initially trained on D_1_ and tested on D_2_ as done previously. Then, the algorithms were retrained on D_1_ with an additional 5% of D_2_, sampled randomly. This process was repeated in 5% intervals, incrementally increasing the amount of D_2_ in the training dataset; the results are displayed in Figure 8.

Figure 8 shows how the accuracies of the models increase as a larger fraction of $D_{2}$ is added to the training set. When none of $D_{2}$ was used to train the models, a 73.0% accuracy was achieved with the DNN in comparison to the ∼33% accuracy obtained with the 1D-CNN and SVMs (as previously described). However, when a larger proportion of $D_{2}$ is used to train the DNN, its accuracy only increases slightly, plateauing at ∼90%. In contrast, although the 1D-CNN performs poorly when β=0, its diagnostic accuracy quickly improves as it gets trained on a larger amount of $D_{2}$, reaching a maximum of 99.1%. The steep increase in accuracy when a small addition of $D_{2}$ is used to train the 1D-CNN is particularly appealing, suggesting that it could perform well for generalization diagnosis, where a small amount of data from a variety of MEAs is used for training.

The SVM models underperform in comparison to the neural networks due to their formulation, whereby the C and ξ parameters allow the misclassification of some faults on the wrong side of the hyperplanes. Therefore, adding a small amount of D_2_ to the SVM training set may have no effect on its performance, since it is fundamentally designed in such a way to allow some outliers to be misclassified. Hence, if data from another MEA is significantly different and causes overlap, the SVM model will treat them as outliers, and the hyperplanes will not be updated. In contrast, the Adam optimizer used in the neural networks will attempt to minimize loss and update their fitting parameters regardless of overlap or noise, enabling them to be influenced by a wider range of data and enhancing their generalization potential. This is directly reflected in their improved accuracy over the SVMs when a small quantity of unseen data is used for training.

The local connectivity and convolution of the 1D-CNN promote its superiority over the other models since they significantly reduce the dimensionality of the AC voltage response input, allowing the model to fit more closely to the training data and achieve higher diagnostic accuracies. Although it was incapable of predicting the SoH of an MEA that was not included in the training set, it responded well to the inclusion of small amounts of unseen training data, attaining an accuracy of 95.1% when only 20% of the unseen data was added to the training set. Moreover, as proposed by Mao et al.,^32^ multi-sensor signals could achieve better diagnostic performances than cell voltages alone; combining the AC voltage response with other diagnostic features could promote the robustness of the framework to detect a wider range of faults, such as flooding and CO poisoning, that were not explored here.

## Discussion

In this paper, a black-box model approach was developed to diagnose PEM fuel cell faults, involving the use of multifrequency Walsh function AC perturbation signals to capture convoluted information on their SoH. The Walsh function was employed to minimize the overall amplitude of the perturbation signals, improving the signal-to-noise ratio while ensuring that the PEFC is not damaged by high amplitude perturbations. The corresponding voltage responses were then used directly as the diagnostic inputs to a DNN, 1D-CNN, linear-SVM, and RBF-SVM to compare their effectiveness at classifying the SoH; all models were able to effectively diagnose normal, drying, and starvation conditions of an individual MEA, with the 1D-CNN and SVMs achieving 100% diagnostic accuracy.

To assess their generalization performance, the models were tested using data generated in the same manner from another identically fabricated MEA. The models were unable to diagnose the SoH of the unseen MEA when trained on the old one, with the DNN achieving the best accuracy of only 72%; even though the data acquisition and manufacture of both MEAs were the same, the voltage responses were too dissimilar to be grouped by the pre-trained algorithms. Conversely, combining the data from both MEAs into one rich dataset yielded superior results, with the 1D-CNN achieving the highest diagnostic accuracy of 99.2%. Furthermore, the 1D-CNN also achieved a better generalization performance when trained with a small proportion of data from the unseen MEA. When 20% of the unseen data was used in training, it attained an accuracy of 95.1% in comparison to the DNN and linear-SVM, which fell short at 83.0% and 66.2%, respectively. Therefore, the parameter sharing and local connectivity of the convolutional layers in the 1D-CNN allowed for higher computational efficiency, reduced errors, and faster convergence, making it the most suitable model architecture for the diagnostic framework. It is expected to be capable of managing a wide range of data from a variety of MEAs while maintaining high accuracy, paving the way for generalized, on-board fault diagnostics.

Looking to the future, a wider range of operating conditions, fault types, and MEAs should be included in the training set to ensure that the model can be generalized to other systems; however, further testing needs to be performed to understand if the model can interpolate/extrapolate for MEAs at unseen conditions to those within the training dataset. As well as different operating conditions, the effect of long-term operation on diagnostic accuracy needs to be explored, since performance degradation over time may reduce, causing the pre-trained model to become redundant. The framework should also be extended to a PEFC stack using multiple voltage responses from different cells simultaneously. This is now feasible given our recent demonstration of multichannel voltage responses^24^ and adapted to diagnose more types of faults; however, both of these improvements will necessitate a larger model with more parameters, making hyperparameter optimization more critical. Although not explored here, the methodology could also be applied to other electrochemical devices, such as lithium-ion batteries for rapid on-line diagnostics in a variety of different applications.

### Limitations of the study

While the proposed diagnostic framework demonstrates high accuracy in controlled settings, several limitations remain. In real-world fuel cell systems, faults often manifest as gradual degradations or soft differences, such as varying degrees of flooding or starvation, rather than the clearly defined fault categories like complete starvation or severe water management issues used in this study. The model has also not been validated on a large batch of fuel cell stacks, which is important for confirming its generalization across different manufacturing variations and long-term operational conditions. Furthermore, the method has not yet been tested on commercial fuel cell systems operating in industrial environments, where complex system interactions and external noise may impact diagnostic accuracy. These limitations must be addressed to fully establish the robustness and practical applicability of the approach.

## Resource availability

### Lead contact

Further information and requests for resources should be directed to and will be fulfilled by the lead contact, Shangwei Zhou (shangwei.zhou.20@ucl.ac.uk).

### Materials availability

This study did not generate new, unique reagents.

### Data and code availability

• Experimental data have been deposited at the Mendeley Data repository and are publicly available as of the date of publication at https://doi.org/10.17632/pn95bsbhv9.1.
• All original code has been deposited at Zenodo and is publicly available at https://doi.org/10.5281/zenodo.16734066 as of the date of publication.
• Any additional information required to reanalyze the data reported in this paper is available from the lead contact upon request.

## Acknowledgments

S.Z. acknowledges the STFC Early Career Research (ST/R006873/1) for funding support. R.J. acknowledges support from the 10.13039/501100000266UK Engineering and Physical Sciences Research Council (EPSRC) - EP/W033321/1.

## Author contributions

Conceptualization, S.Z.; data curation, G.D'S., E.M., and S.Z.; formal analysis, G.D'S. and E.M.; investigation, G.D'S. and E.M.; methodology, G.D'S. and E.M.; visualization, G.D'S., E.M., and S.Z.; writing – original draft, G.D'S., E.M., and S.Z.; writing – review and editing, G.D'S., E.M., R.J., and S.Z.; resources, R.J. and S.Z.; funding acquisition, R.J.; supervision, R.J. and S.Z.; project administration, S.Z.

## Declaration of interests

The authors declare no competing interests.

## STAR★Methods

### Key resources table

**Table:** 

REAGENT or RESOURCE	SOURCE	IDENTIFIER
**Deposited data**		

Experimental data	This paper	Mendeley Data: https://doi.org/10.17632/pn95bsbhv9.1

**Software and algorithms**		

Python 3.10.2	Python Software Foundation	https://www.python.org/
Gamry Framework	Gamry Framework	https://www.gamry.com/
Analysis Code	This paper	Zenodo: https://doi.org/10.5281/zenodo.16734066

**Other**		

850e Fuel Cell Test System	Scribner Associates	https://www.scribner.com/
Reference 3000	Gamry Framework	https://www.gamry.com/
Gas diffusion electrodes	HyPlat	https://www.hyplat.com/
211 membrane	Nafion	https://www.nafion.com/en/

### Method details

The MEA seen in Figure S1 was fabricated in-house, using gas diffusion electrodes (GDEs) with platinum loadings of 0.4 mg cm-2 (HyPlat, South Africa). These employed 250 μm thick H23C9 carbon paper and MPLs (Freudenberg, Germany) with hydrophobic treatment to facilitate water removal. The GDEs were cut into two 5 × 5 cm squares and sandwiched between an 8 × 8 cm square of Nafion™ 211 membrane (DuPont, USA), with a thickness of 25.4 μm. Using a hot press (Carver Inc., USA), the GDEs and membrane were pressed together for 3 minutes at 140 °C, with a clamping pressure of 2.75 MPa to bond the layers together and form the MEA.^33^

The MEA was sealed with a torque of 4 Nm in the PEFC test fixture (Scribner Associates, USA) between two graphite flow fields, consisting of 17 serpentine passes with channel widths and depths of 1 mm. The PEFC was then installed into an 850e test system (Scribner Associates, USA), undergoing a conditioning cycle to ‘break-in’ the MEA before the polarisation curves and diagnostic tests were carried out.^34^

Diagnostic tests at three different health states (normal, reactant starvation, drying) were performed at a current density of 400 mA cm^-2^. To induce starvation, the cathode-side stoichiometry was reduced from +3.0 to +1.5, reducing the amount of oxygen available for the reaction. Although a stoichiometry of +1.5 indicates 50% more oxygen than is theoretically required, it is likely to cause some starvation, particularly on a local level. To induce drying, the inlet temperatures of the anode and cathode were reduced to ∼40°C (while maintaining a cell temperature of 60°C). This reduced the amount of water entering the system.

### Quantification and statistical analysis

There are no quantification or statistical analyses to include in this study.
