# Supplementary material for: Generalized fault diagnostics of polymer electrolyte fuel cells using machine learning
Source: iScience. 2025 Aug 13;28(9):113350. doi: 10.1016/j.isci.2025.113350 (PMC12446379; doi:10.1016/j.isci.2025.113350)
Supplement: Document S1. Figures S1 and S2 and Data S1 [file mmc1.pdf]

iScience, Volume *28*

Supplemental information

Generalized fault diagnostics of polymer electrolyte fuel cells using machine learning

Greg D'Silva, Eashaal Mahmood, Rhodri Jervis, and Shangwei Zhou

Supplemental Figures

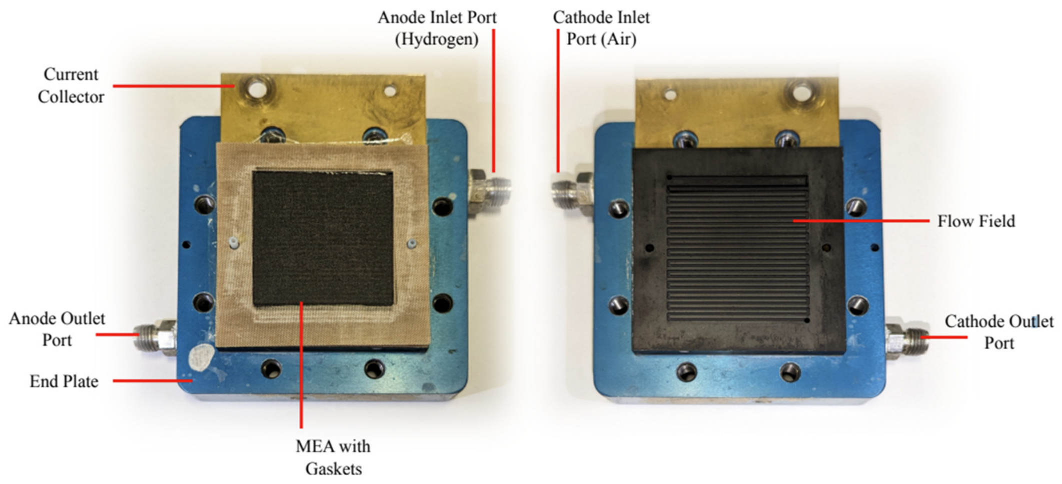

Figure S1. Internal schematics of the Scribner fuel cell test fixture.

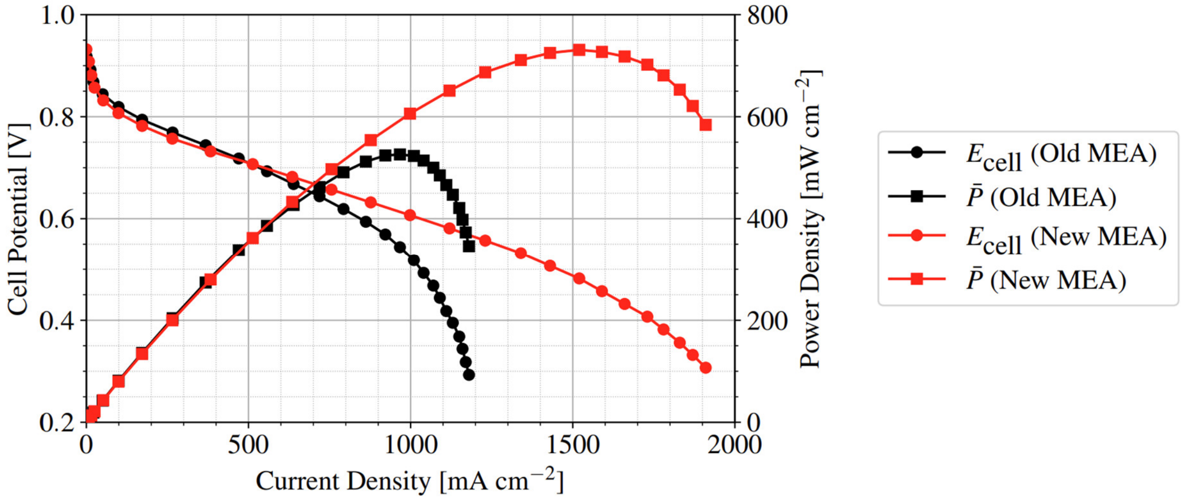

Figure S2. Polarisation and power curves for the original MEA (black) and new MEA (red) before carrying out the fault diagnostic procedures. The new MEA had a better initial performance compared to the old MEA.

Data S1: Diagnosis algorithms

Dense Neural Network (DNN)

The objective of the DNN is to learn a set of parameters $\boldsymbol{\Theta}$ that map the input $\boldsymbol{X}$ (voltage response) to the output $\boldsymbol{y}$ (SoH: normal, drying, starvation) according to Equation % [S1]:

$$\boldsymbol{y} = F(\boldsymbol{X}|\boldsymbol{\Theta}) = f_{D,L}\big(f_{D,L-1}\big(\dots f_{D,2}\big(f_{D,1}(\boldsymbol{X}|\boldsymbol{\Theta}_1)\big|\boldsymbol{\Theta}_2\big)|\boldsymbol{\Theta}_{L-1}\big)\big|\boldsymbol{\Theta}_L\big), \tag{S1}$$

where L is the number of layers. The ith dense layer $f_{D,i}$ can be expressed as:

$$f_{D,i}(\boldsymbol{X}_{i-1}|\boldsymbol{\Theta}_i) = \sigma(\boldsymbol{W} \cdot \boldsymbol{X}_{i-1} + \boldsymbol{b}), \qquad \boldsymbol{\Theta} \in \{\boldsymbol{W}, \boldsymbol{b}\}, \tag{S2}$$

where $\boldsymbol{W}$ and $\boldsymbol{b}$ are the weight and bias matrices respectively, representing the fitting parameters of the neurons, and σ is the activation function to introduce nonlinearity into the network. For this, the ReLU function was used [S2]:

$$\text{ReLU}(x) = \max(0, x) = \begin{cases} 0, & x < 0 \\ x, & x \geq 0 \end{cases} \tag{S3}$$

The softmax classifier was used to calculate the output probability distribution according to:

$$\text{softmax}(x_i) = \frac{\exp(x_i)}{\sum_{j=1}^{n} \exp(x_j)}, \tag{S4}$$

where x_i is the output of ith neuron at the final layer and n is the size of the output layer. The Adam optimiser was used to minimise the cross-entropic loss during backpropagation [S3], [S4]:

$$\text{Loss} = -\sum_{i=1}^{n} t_i \log(p_i), \tag{S5}$$

where t_i is the true label and p_i is the softmax probability for the ith output neuron. Further information on the theory behind DNNs is explained by Looney [S5].

1-Dimensional Convolutional Neural Network (1D-CNN)

The structure of the 1D-CNN differs from the DNN due the addition of convolutional layers which seek to uncover correlation within the input. The ith convolutional layer $f_{C,i}$ can be expressed as [S6]:

$$f_{C,i}(\boldsymbol{X}_{i-1}|\boldsymbol{\Theta}) = \sigma(\boldsymbol{W} \otimes \boldsymbol{X}_{i-1} + \boldsymbol{b}), \qquad \boldsymbol{\Theta} \in \{\boldsymbol{W}, \boldsymbol{b}\}, \tag{S6}$$

where $\otimes$ is the convolution operation in which a filter slides over the input layer and computes the dot product at each position, generating a feature map that captures spatial correlations. Subsampling or ‘pooling’ layers were also implemented to reduce the dimensionality of the feature maps and prevent overfitting. Lawrence et al. outline further details on CNN theory [S7].

Support Vector Machine

SVM aims to find the optimal hyperplane(s) that best separates the input data x_j ($j = 1, \ldots, m$) into different classes. The algorithm works to maximise the margin between different classes while minimising the classification error. Since the PEFC diagnosis involves three SoH classes, multiclass SVM is required. The ith SVM solves the following optimisation problem [S8]:

$$minimise: \quad \frac{1}{2}\left|\left|\boldsymbol{w}^i\right|\right|^2 + C\sum_{j=1}^{m}\xi_j^i\left(\boldsymbol{w}^i\right)^T \tag{S7}$$

$$subject\ to: \quad \left(\boldsymbol{w}^i\right)^T\phi\left(\boldsymbol{x}_j\right) + b^i \geq 1 - \xi_j^i, \qquad if\ y_j = i, \tag{S8}$$

$$\left(\boldsymbol{w}^i\right)^T\phi\left(\boldsymbol{x}_j\right) + b^i \leq -1 + \xi_j^i, \qquad if\ y_j \neq i, \tag{S9}$$

$$\xi_j^i \geq 0, \qquad j = 1, \ldots, m \tag{S10}$$

where $\boldsymbol{w}$ is the perpendicular distances between the features $\boldsymbol{x}_i$ and the hyperplane, C and ξ_j are the error penalty and slack variables respectively which serve to penalise features classified on the wrong side of the hyperplane, b is a bias term, and $\phi(\boldsymbol{x}_j)$ is the function to map the input into a higher-dimensional space to classify non-linearly separable data. The mapping is performed by kernel functions k; the linear k_L and radial basis function k_{RBF} kernels were tested on the dataset [S9]:

$$k_L\left(\boldsymbol{x}, \boldsymbol{x}_j\right) = \phi(\boldsymbol{x})^T\phi\left(\boldsymbol{x}_j\right) = \boldsymbol{x}^T\boldsymbol{x}_j \tag{S11}$$

$$k_{RBF}\left(\boldsymbol{x}, \boldsymbol{x}_j\right) = \phi(\boldsymbol{x})^T\phi\left(\boldsymbol{x}_j\right) = \exp\left(-\gamma\left\|\boldsymbol{x} - \boldsymbol{x}_j\right\|^2\right), \qquad \gamma > 0, \tag{S12}$$

where γ defines how much influence a single training feature has. Further information on SVM theory is outlined by Suthaharan [S10].

Supplimental references

[S1] S. Abdoli, P. Cardinal, and A. Lameiras Koerich, “End-to-end environmental sound classification using a 1D convolutional neural network,” *Expert Syst Appl*, vol. 136, pp. 252–263, Dec. 2019.

[S2] B. Ding, H. Qian, and J. Zhou, “Activation functions and their characteristics in deep neural networks,” in *2018 Chinese Control And Decision Conference (CCDC)*, Jun. 2018, pp. 1836–1841–1836–1841.

[S3] D. P. Kingma and J. Ba, “Adam: A Method for Stochastic Optimization,” *arXiv Preprint arXiv:1412.6980*, Jan. 2017.

[S4] A. Mao, M. Mohri, and Y. Zhong, “Cross-Entropy Loss Functions: Theoretical Analysis and Applications,” in *Proceedings of the 40th International Conference on Machine Learning*, A. Krause, E. Brunskill, K. Cho, B. Engelhardt, S. Sabato, and J. Scarlett, Eds., PMLR, Jun. 2023, pp. 23803–23828–23803–23828.

[S5] C. G. Looney, *Pattern Recognition using Neural Networks: Theory and Algorithms for Engineers and Scientists*. Oxford University Press, Inc., 1997.

[S6] S. Abdoli, P. Cardinal, and A. Lameiras Koerich, “End-to-end environmental sound classification using a 1D convolutional neural network,” *Expert Syst Appl*, vol. 136, pp. 252–263–252–263.

[S7] S. Lawrence, C. L. Giles, A. C. Tsoi, and A. D. Back, “Face recognition: a convolutional neural-network approach,” *IEEE Trans Neural Netw*, vol. 8, no. 1, pp. 98–113–98–113, Jan. 1997.

[S8] A. Widodo and B.-S. Yang, “Support vector machine in machine condition monitoring and fault diagnosis,” *Mech Syst Signal Process*, vol. 21, no. 6, pp. 2560–2574–2560–2574, Aug. 2007.

[S9] A. Patle and D. S. Chouhan, “SVM kernel functions for classification,” in *2013 International Conference on Advances in Technology and Engineering (ICATE)*, Jan. 2013, pp. 1–9–1–9.

[S10] S. Suthaharan, “Support Vector Machine,” in *Machine Learning Models and Algorithms for Big Data Classification*, S. Suthaharan, Ed., Springer US, 2016, pp. 207–235–207–235.
